# The Potential Role of the Fat–Glandular Interface (FGI) in Breast Carcinogenesis: Results from an Ultrasound Tomography (UST) Study

**DOI:** 10.3390/jcm10235615

**Published:** 2021-11-29

**Authors:** Nebojsa Duric, Mark Sak, Peter J. Littrup

**Affiliations:** 1 Department of Imaging Sciences, University of Rochester, Rochester, NY 14642, USA; 2 Delphinus Medical Technologies Inc., Novi, MI 48374, USA; plittrup@delphinusmt.com; 3 School of Medicine, Wayne State University, Detroit, MI 48202, USA; msak@delphinusmt.com

**Keywords:** breast cancer, ultrasound tomography, fibroglandular–fat interface, fibroglandular tissue composition, carcinogenesis

## Abstract

This study explored the relationship between the extent of the fat–glandular interface (FGI) and the presence of malignant vs. benign lesions. Two hundred and eight patients were scanned with ultrasound tomography (UST) as part of a Health Insurance Portability and Accountability Act (HIPAA)-compliant study. Segmentation of the sound speed images, employing the k-means clustering method, was used to help define the extent of the FGI for each patient. The metric, α, was defined as the surface area to volume ratio of the segmented fibroglandular volume and its mean value across patients was determined for cancers, fibroadenomas and cysts. ANOVA tests were used to assess significance. The means and standard deviations of α for cancers, fibroadenomas and cysts were found to be 4.0 ± 2.0 cm^−1^, 3.1 ± 1.7 cm^−1^ and 2.3 ± 0.9 cm^−1^, respectively. The differences were statistically significant (*p* < 0.001). The separation between the groups increased when α was measured on only the image slice where the finding was most prominent, with values for cancers, fibroadenomas and cysts of 5.4 ± 3.6 cm^−1^, 3.6 ± 2.3 cm^−1^ and 2.4 ± 1.5 cm^−1^, respectively. Of the three types of masses studied, cancer was associated with the most extensive FGIs, suggesting a potential role for the FGI in carcinogenesis, a subject for future studies.

## 1. Introduction

In breast imaging, the fat–glandular interface (FGI) is defined as the boundary that separates the dense glandular tissue from the subcutaneous fat of the breast. Previously reported studies aimed to evaluate preferred locations and properties of common breast masses by magnetic resonance (MR) imaging [1,2] and more recently by ultrasound tomography (UST) [3], in relation to the FGI. In the latter study, it was found that 93% (169/181) of cancers were located at the FGI vs. 69% (129/188) of fibroadenomas and 40% (69/176) of cysts. All studies suggested that cancers are more likely to be found at the FGI compared to the most common benign masses. However, a quantifiable parameter describing the diversity of the FGI appearances for benign and malignant masses has not been developed. Such a parameter would provide the impetus for additional risk evaluations.

The association of breast cancer with the FGI is not obvious in digital mammography (DM) and digital breast tomosynthesis (DBT) images due to breast compression and tissue overlap, which mask the FGI. Similar considerations apply to automated breast ultrasound (ABUS). However, with breast MR and UST, where the breasts are imaged in a prone uncompressed position, the FGI becomes more apparent. The emerging field of ultrasound tomography (UST) provides a further advantage because, unlike ABUS, it does not distort the breast and unlike MRI, it does not require contrast injection for optimal cancer detection. Any potential use of UST for screening would capitalize on these findings to support targeted searches and automated analyses to optimize clinical workflow and reduce call back rates. 

UST is an emerging imaging technique that has been specifically adapted for breast imaging [4,5,6,7,8,9,10,11,12,13,14,15,16,17,18,19]. It provides whole breast and focal mass evaluation by combining reflection signal acquisition with quantitative transmission properties of sound speed (SS) and attenuation (ATT). UST offers the quantitative multi-parametric evaluation of breast tissues that are scanned and displayed in its highest resolution coronal plane. SS imaging provides high contrast between fat and denser parenchyma/stromal tissues and shows a good correlation with MR parenchymal patterns, as well as the volumetric assessment of the entire breast [14,15]. SS has also been shown to be a stronger breast cancer (BC) risk factor than mammographic density [12]. The development of an objective parameter describing FGI diversity may thus contribute to risk assessments related to SS and breast density.

The specific UST device used in this study was SoftVue, recently approved by the FDA for breast cancer screening (Delphinus Medical Technologies, Novi, MI, USA). A SoftVue scan is operator-independent and covers the entire volume of the breast, up to the axilla. The patient lies prone on a table that houses a water bath in which the breast is pendant during scanning. A ring-shaped sensor surrounds the breast inside the water bath and scans the whole breast from nipple to chest wall in approximately two minutes, providing a stack of 2.5 mm thick coronal images. SoftVue’s operating characteristics include a frequency range of 1–3 MHz and a spatial resolution of 0.75 mm in the coronal acquisition plane and 2.5 mm out of plane. The coronal image stacks are co-registered for their different presentations, providing clinical image stacks of reflection and two stacks of transmission images consisting of sound speed and stiffness. Thumbnail axial and sagittal reconstructions provide 3D localization, along with the sequential coronal image review. Furthermore, the patient’s position matches the appropriate clock position, and an external calibrated encoder provides the anterior–posterior (AP) distance relative to the nipple. For the purposes of this study, it is the sound speed image stacks that represent the volume of the breast to be interpreted. 

In this study, we use UST to build on previous work [3] by developing a quantitative metric that characterizes the exposure of “at risk” fibroglandular tissues to the potential cancer-initiating hormonal effects of fat at the FGI. We then apply that metric to the data to explore relationships between FGI exposure and the presence of malignant vs. benign lesions. Such a relationship would provide quantitative constraints on biological models for carcinogenesis and formation of benign masses. 

## 2. Material and Methods

### 2.1. Subjects and Masses

Data were obtained from scans of patients who were recruited to the diagnostic arm of a HIPAA-compliant, multi-arm, multi-center trial of SoftVue UST (Clinicaltrials.gov—NCT#02977247: Delphinus SoftVue Prospective Case Collection—ARM 2 (SV PCC ARM2)). Patients were eligible to receive SoftVue imaging as part of their clinical visit for evaluation of a palpable or mammographic abnormality. Informed consent was thus obtained from all women within this observational cohort study, whereby the main inclusion criterion was their willingness to participate in a SoftVue scan during their clinical visit. Notable exclusion criteria were age <18 years, body weight >350 pounds (i.e., the SoftVue scanning table projected limit), inability to give informed consent, inability to lie prone on the UST table, and any open sores or wounds on the breast precluding immersion into the UST water bath. Water within the SoftVue scanning tank was exchanged between patients and sanitized with ProTex (Parker laboratories Inc., Fairfield, NJ, USA). For this study, a consecutive data set was used, with all patients having UST scan dates in the period April 2017–October 2018, with the same version of the SoftVue unit and associated reconstruction algorithms used across all centers of the trial. Data from the other arm of the multi-center study using UST for dense breast screening (i.e., NCT03257839: Delphinus SoftVue Prospective Case Collection—ARM 1 (SV PCC ARM1)) are not reported here. 

All identified masses were biopsy-confirmed by subsequent or prior histology, unless considered characteristic cysts by hand-held ultrasound criteria. All complicated cysts underwent aspiration with cytologic confirmation. Some women had more than one mass in each or both breasts. Masses were grouped into the main categories of cancer, fibroadenomas and cysts. Breasts with multiple unique findings were grouped according to their most critical finding: cancers, then fibroadenomas, then cysts. Breast density was obtained as part of the study via categorization of the mammogram into one of the four breast density categories.

### 2.2. Segmentation of Images 

Segmentation of sound speed images was used to separate the tissue into categories of fibroglandular (i.e., mammographically “dense”) or fatty by the use of K-means clustering techniques in the open-source package ImageJ [19]. The segmentation was applied to all images in a stack except near the chest wall or nipple. The presence of the chest wall would incorrectly be labeled as fibroglandular tissue and the gel pad used to secure the nipple in the proper imaging position would cause inconsistent masking and segmentation. Only the breast containing the studied mass was analyzed. The result of the segmentation produced a mask of the fibroglandular regions of the breast and represented the first step in determining the FGI, as described below.

### 2.3. Surface Area to Volume Ratio as a Metric for FGI Exposure Extent

The segmented fibroglandular mask of the sound speed image was used to develop a metric for the extent of the FGI. To limit the effect of small and irregularly shaped areas, all fibroglandular regions smaller than 50 pixels were first removed. On each slice, *i_j_*, the circumference of the fibroglandular component, *c_i_*, was measured in pixels and converted to a length in cm. The area of the fibroglandular component was also measured in squared pixels and then converted to an area in cm^2^. The slice thickness was then multiplied by these measurements to obtain, respectively, the surface area (SA) and volume (V) of the slice. The extent of the FGI for each slice could then be calculated as α = *SA*/*V*, in units of cm^−1^. A value of α could also be measured for the entire volume of the breast by simply summing the perimeter of all regions on all slices and by summing the area of the region on all slices. Since both the surface area and volume measures involve a product with the slice thickness, when α is calculated the slice thickness is cancelled out. The α values were then binned according to whether the patients had a cancer, fibroadenoma or cyst. The distribution of α values was then determined within each bin. 

ANOVA tests were then applied to determine the significance of any differences in the mean values of the three distributions. A post hoc pairwise comparison was also performed on any statistically significant ANOVA result using a Tukey HSD test. In the unlikely case where the data were not normally distributed, a Kruskal–Wallis (KW) test was also performed and compared to the results of the ANOVA test.

## 3. Results

### 3.1. Subjects and Masses

**Figure 1:**
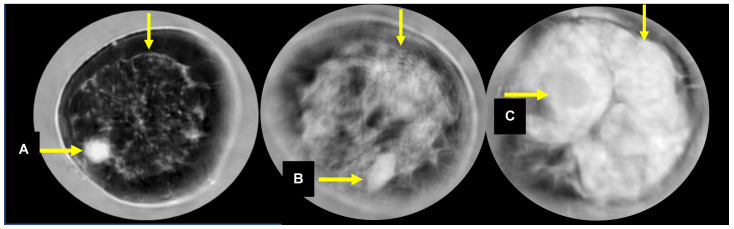
Sound speed images of a cancer (**A**), fibroadenoma (**B**) and cyst (**C**). The unmarked arrows point to the FGI.

The average age for participants in this study was 48.9 years (standard deviation = 11.6 years, range 18–82 years). The retrieved data sets represent 241 individual breasts from 208 diagnostic patients with 300 masses, including cancer and benign masses, as noted in Table 1. There were 1.4 masses per woman (300/208) or 1.2 (300/241) masses per SoftVue scanned breast. The average total breast volume was 737 mL, and the average tumor volume was 1.1 mL. Over 90% of patients had heterogeneously or extremely dense breasts by mammography (i.e., *N* = 135 (64.9%), or *N* = 55 (26.4%), respectively). Patients with suspicious masses were also included from women with scattered breast density (*N* = 18, or 8.7%). This clinical cohort of women with breast masses is skewed toward the higher mammographic breast densities because the multi-center study was aimed at assessing UST performance in women with dense breasts. A breakdown of the findings by histology was available, but individual counts for most findings were too low to allow for reliable differentiation. In the case of the “other benign” category, 24 masses were not used in the study because of the unrelated subtypes and small numbers. The subsequent results are presented for 276 masses representing the categories of cancer, fibroadenoma and cyst. An example of a sound speed image of a cancer associated with the FGI is shown in Figure 1. 

### 3.2. Breast Density Distribution by Category

The original study was designed to image women with dense breasts. Breast density was obtained from patient records corresponding to their mammograms at the time of entry into the study. Most cases fell into the c (*N* = 135) and d (*N* = 55) breast density categories. A small number of b density cases (*N* = 18) were also imaged.

### 3.3. Distribution of α Parameter by Mass Type

**Figure 2:**
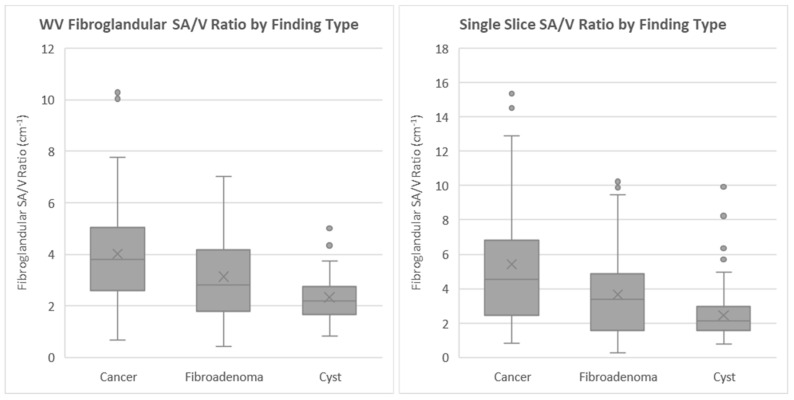
Distribution of α values using the volume method (**left**) and using the single-slice method (**right**).

The frequency distribution of α is shown for each mass type (cancer, fibroadenoma and cyst) in Figure 2.

The mean values and standard deviations of α for each mass type are summarized in Table 2. The ANOVA tests on α distribution differences indicate that the three distributions are significantly different (*p* < 0.001). The results of the post hoc pairwise analysis are shown in Table 3 and indicate that there was a statistically significant difference in the mean values of α between each grouping. The similar results of the KW and ANOVA tests indicate that the findings are robust to any potential violations of normality in the data.

## 4. Discussion

We defined the α parameter as the surface area to volume ratio of the FGI, to be used as a quantitative measure of FGI extent. The metric was applied to image data containing cancers, fibroadenomas and cysts. Distributions of α were then determined for all three mass types. While there is considerable overlap in the values of α among all three mass types, there is a steady decrease in the average value of α in a sequence from cancer to fibroadenomas to cysts. This is true when α is measured over the whole volume or when measured on the single slice where the mass is best defined. The ANOVA tests indicate that there is a statistically significant difference in the mean values for each finding type, and the post hoc pairwise analysis indicates that there is a statistically significant difference in the mean values between each possible grouping. 

### 4.1. Trends in the α Distributions

Previous studies reported the frequency of cancer vs. benign mass occurrence at the FGA (either yes or no) but did not explore the relationship between the amount of FGI and the presence of cancer vs. benign masses. The α parameter trends observed in this study suggest that the presence of cancer is dependent not just on the existence of the FGI but also on the amount of FGI. Furthermore, this observation held when measuring the α value over the entire breast and on the individual slice containing the finding. In fact, the separation in the mean α values was greater when measuring the individual slice. The better separation obtained with the single-slice method might have two possible explanations, one tied to image acquisition and the other to biology. The UST image represents a volume with asymmetric voxels because the coronal plane resolution is 0.75 mm but the slice thickness is 2.5 mm so that the ability to segment the FGI in the non-coronal plane is degraded relative to a single slice that does not suffer from this degradation and potentially yields a more accurate value of α. A second possibility is that mass formation is regulated more by the local properties of the FGI vs. its global, whole breast properties. The biological role of the FGI is discussed further below.

### 4.2. Implication for Breast Cancer Risk

It is now well established that cancer incidence is greatest in women with the densest breasts. At the same time, we know that the FGI also plays a role, suggesting that additional factors may be at play. The first such factor is location, the observed tendency of cancers to be at the FGI and the second, the tendency for more extensive FGIs, possibly offering greater exposure of the “at risk” fibroglandular tissue to potential endocrine influences of the adjacent fat. The extent of the FGI is dependent on its surface area averaged over the volume of the breast. Thus, for a given breast size, the more irregular the surface, the greater the area of the FGI, driving the extent of the FGI to higher α values. Thus, the complexity of breast parenchyma, expressed here as α, may be an additional factor that could potentially contribute to breast cancer risk. 

### 4.3. Implication for Carcinogenesis

The qualitative and quantitative associations of cancer with the FGI may have interesting biological and clinical implications that would justify further follow-up work. From a whole breast perspective, defining the preferential origin of cancer at the FGI may better explain the fact that only 20% of cancers occur in the quadrant with the greatest density percentage [3]. Each quadrant’s cancer risk may relate more to the actual surface area of the FGI within the quadrant, while the fat-related biological effects of cancer initiation at the FGI may arise more from random genetics occurring within susceptible adjacent fibroglandular tissue of any quadrant. Our quantitative analyses confirmed that cancers were more likely to straddle fat and dense tissue, but further pathological analyses are needed to better understand biological changes at the FGI. In the future, the quantitative information presented here may be further utilized to better understand the complex biology of adipocytes, adipokines and cancers cells near the FGI.

As we previously noted [3], fat cells, or adipocytes, and their associated hormonal secretions, or adipokines, play an important role in breast cancer initiation and growth, while also mediating blood pressure, reproductive function, appetite, glucose homeostasis, angiogenesis and immune function [20,21,22,23,24]. An adipokine, leptin, has been implicated in breast cancer initiation via aromatase expression when the balance tips toward an excessive pro-inflammatory state [22,23,24]. Cancer cell lines can show marked tumor growth in the presence of cancer-associated adipocytes and adipocyte-derived fibroblasts, contributing to breast cancer progression. Complex peritumoral stromal processes may thus correspond to the noted hyaluronan deposition and peritumoral apparent diffusion coefficient (ADC) values by breast MRI [25,26], as well as stiff peritumoral rims noted with shear wave elastography [27,28]. The FGI thus describes the fibroglandular boundaries with the subcutaneous adipose layer, which may represent the largest endocrine source for breast cancer origin and growth [22]. 

A possible interpretation of our findings is that the parameter α is a measure of the relative exposure of the “at risk” cells within the terminal ductal lobular unit (TDLU) to the hormonal influences of adjacent adipocytes. The complex origin of most breast cancers may thus be a combination of the genetic propensity (i.e., BRCA1/2 status) in conjunction with the local hormonal milieu that may be subsequently impacted by environmental factors, such as diet and/or obesity. The resultant impact of cancerogenic adipokines upon adjacent TDLUs may thus be dependent upon the exposed surface area of the breast volume to these interactions, analogous to intestinal absorption of dietary nutrients relative to the greatly increased surface area per unit volume afforded by intestinal villi. Similar to the malabsorption that occurs with atrophy of intestinal villi that commonly occur with celiac disease (i.e., gluten sensitive enteropathy), a high-risk breast TDLU may not experience cancer initiation as frequently from low hormonal exposure in a patient with a low FGI α. Thus, cancer initiation and its development into an observable tumor may well be mediated by the FGI, as its association with the FGI would suggest.

However, further explanation is needed to account for the strong trends in α which is a ratio of FGI area to breast volume, as opposed to the FGI surface area alone. One plausible explanation has its parallels with the known association between breast cancer risk and mammographic density, where the driving factor is not the amount of dense tissue but rather the relative amount of dense tissue within a breast. Thinking of the breast as a system, the probability of a random seeding of cancer occurring in dense tissue only depends on the amount of dense tissue within that breast, in other words, the percentage of dense tissue. Thus, for a given percentage of dense tissue, it does not matter if the breast is large or small, the probability of the cancer initiating in dense tissue is the same. By analogy, our finding that the presence of cancer depends on the relative, rather than absolute, amount of FGI is similarly plausible. Furthermore, the association with the FGI suggests (i) that the initiation of cancer may well take place at the surface of the dense tissue that represents the FGI and (ii) that its subsequent growth requires the presence of adjacent fat. Statistically, the opportunity for that growth would then depend on the areas of the available FGI, with larger areas (for a given breast size) representing greater opportunities and a higher likelihood of development. In such a model, the association of breast cancer risk with breast density can be explained as a more specific association with the FGI since, generally, a larger percentage of dense tissue translates to a more extensive FGI. 

### 4.4. Weaknesses

This study was limited to comparing cancer to benign masses in the context of the FGI. It does not, therefore, provide insight into population-based FGI differences since it did not compare FGI distributions of cancer patients against those with no discernable disease (i.e., normal). Furthermore, this study is secondary to the main aim of the parent UST study and the patient population selected does not conform to proper patient modeling concerns. Inherent biases in the data set may be present and have not been accounted for. A planned future study will address these shortcomings. 

The measurement of the surface area of the fibroglandular region required a measurement of the perimeter of this area on a series of images. This perimeter measurement is an example of the coastline paradox wherein the actual perimeter surrounding the fibroglandular area does not have a well-defined length. The length that is measured depends on the length of the ruler used, which in this case depends on the resolution of the image. As the resolution of the image increases, the effective length of the ruler decreases leading to the measured surface area actually increasing while the volume of tissue remains largely the same. Therefore, the absolute value of the measurements of α should not be considered definitive. However, the relative values between images and overall trends between finding types would likely remain consistent providing that consistent resolution images are used for all measurements.

## 5. Conclusions

Of the three types of masses studied, cancer is most strongly associated with the extent of the FGI, with cysts associating most strongly with the least extensive FGIs. Fibroadenomas were most strongly associated with FGIs of intermediate extent. These results suggest a strong role for the FGI in carcinogenesis and cancer progression. A possible biological interpretation of our findings is that the extent of the FGI is a measure of the relative exposure of the “at risk” cells within the terminal ductal lobular unit (TDLU) to the hormonal influences of adjacent adipocytes. Future studies with larger cohorts and a wider range of breast densities will further illuminate the role of the FGI. 

## Figures and Tables

**Table 1:** Frequency of mass types in this study. A total of 300 masses were evaluated.

Mass Histology	Count (*N*)
Cancer	80
Fibroadenoma	105
Cyst	91
Other benign	24
Subtypes: Containing fibrosis	
Fibrocystic change	
Granulomatous Mastitis	
Total	300

**Table 2:** Characteristics of the α distribution for cancers, fibroadenomas and cysts measured over both the whole breast volume and the single slice containing the finding. Means and standard deviations are shown from the whole volume and single slice analyses.

Mass Type	Whole Volume Mean α (cm^−1^)	St. Dev	Single Slice α (cm^−1^)	St. Dev
Cancer	4.0	2.0	5.4	3.6
Fibroadenoma	3.1	1.7	3.7	2.3
Cyst	2.3	0.9	2.4	1.5
ANOVA test	*p* < 0.001		*p* < 0.001	
KW test	*p* < 0.001		*p* < 0.001	

**Table 3:** Results of the Tukey HSD post hoc test for pairwise comparisons of the whole volume and single-slice α value.

Comparison	Whole Volume Measurements	Single Slice Measurements
Cancer–Cyst	*p* < 0.001	*p* < 0.001
Cancer–Fibroadenoma	*p* < 0.001	*p* < 0.001
Fibroadenoma–Cyst	*p* = 0.001	*p* = 0.003

## Data Availability

The data presented in this study are available in article.
